# Effect of the uronic acid composition of alginate in alginate/collagen hybrid hydrogel on chondrocyte behavior

**DOI:** 10.3389/fbioe.2023.1118975

**Published:** 2023-03-07

**Authors:** Guoshuang Zheng, Chundong Xue, Fang Cao, Minghui Hu, Maoyuan Li, Hui Xie, Weiting Yu, Dewei Zhao

**Affiliations:** ^1^ Laboratory of Orthopedics, Affiliated Zhongshan Hospital of Dalian University, Dalian, China; ^2^ National-Local Joint Engineering Laboratory for the Development of Orthopedic Implant Materials, Dalian, China; ^3^ Department of Orthopedics, Affiliated Zhongshan Hospital of Dalian University, Dalian, China

**Keywords:** chondrocyte behavior, alginate, guluronate/mannuronate acid ratio (G/M ratio), hydrogel, mechanical properties, cartilage tissue engineering

## Abstract

**Introduction:** Developing a culture system that can effectively maintain chondrocyte phenotype and functionalization is a promising strategy for cartilage repair.

**Methods:** An alginate/collagen (ALG/COL) hybrid hydrogel using different guluronate/mannuronate acid ratio (G/M ratio) of alginates (a G/M ratio of 64/36 and a G/M ratio of 34/66) with collagen was developed. The effects of G/M ratios on the properties of hydrogels and their effects on the chondrocytes behaviors were evaluated.

**Results:** The results showed that the mechanical stiffness of the hydrogel was significantly affected by the G/M ratios of alginate. Chondrocytes cultured on Mid-G/M hydrogels exhibited better viability and phenotype preservation. Moreover, RT-qPCR analysis showed that the expression of cartilage-specific genes, including SOX9, COL2, and aggrecan was increased while the expression of RAC and ROCK1 was decreased in chondrocytes cultured on Mid-G/M hydrogels.

**Conclusion:** These findings demonstrated that Mid-G/M hydrogels provided suitable matrix conditions for cultivating chondrocytes and may be useful in cartilage tissue engineering. More importantly, the results indicated the importance of taking alginate G/M ratios into account when designing alginate-based composite materials for cartilage tissue engineering.

## Graphical Abstract

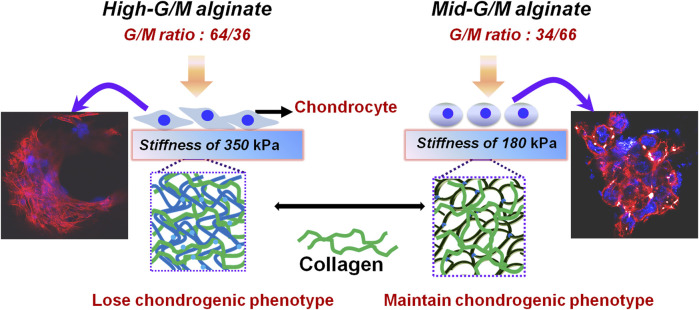

## 1 Introduction

Articular cartilage defects can lead to pain and eventually lead to osteoarthritis, which remains a major challenge in orthopedics worldwide (Gan et al., 2019). Articular cartilage has no neural tissue and no blood supply, and its ability to repair itself is poor. The difficulty in regenerating cartilage is ascribed to the inability to stimulate the generation of new cells or form a chondrogenic matrix (Xingchen et al., 2018; Fu et al., 2020). Autologous chondrocyte implantation has been one of the most promising treatments for cartilage regeneration in recent years. However, the use of autologous chondrocytes requires the *in vitro* expansion of chondrocytes prior to implantation, requiring long expansion times and multiple passages in monolayer cultures, often resulting in a loss of their typical phenotype and function (Jin et al., 2014; Mahapatra et al., 2016; Karim and Hall, 2017). As a result, the cartilage defect will be filled with fibrous cartilage and an inability to regain the function of normal hyaline cartilage (Murphy et al., 2020). Therefore, chondrocyte dedifferentiation is a major challenge for restoring normal cartilage function. To address this issue, well-designed cultural systems that facilitate stable phenotyping and definite functionalization of chondrocytes should be established.

**FIGURE 1:**
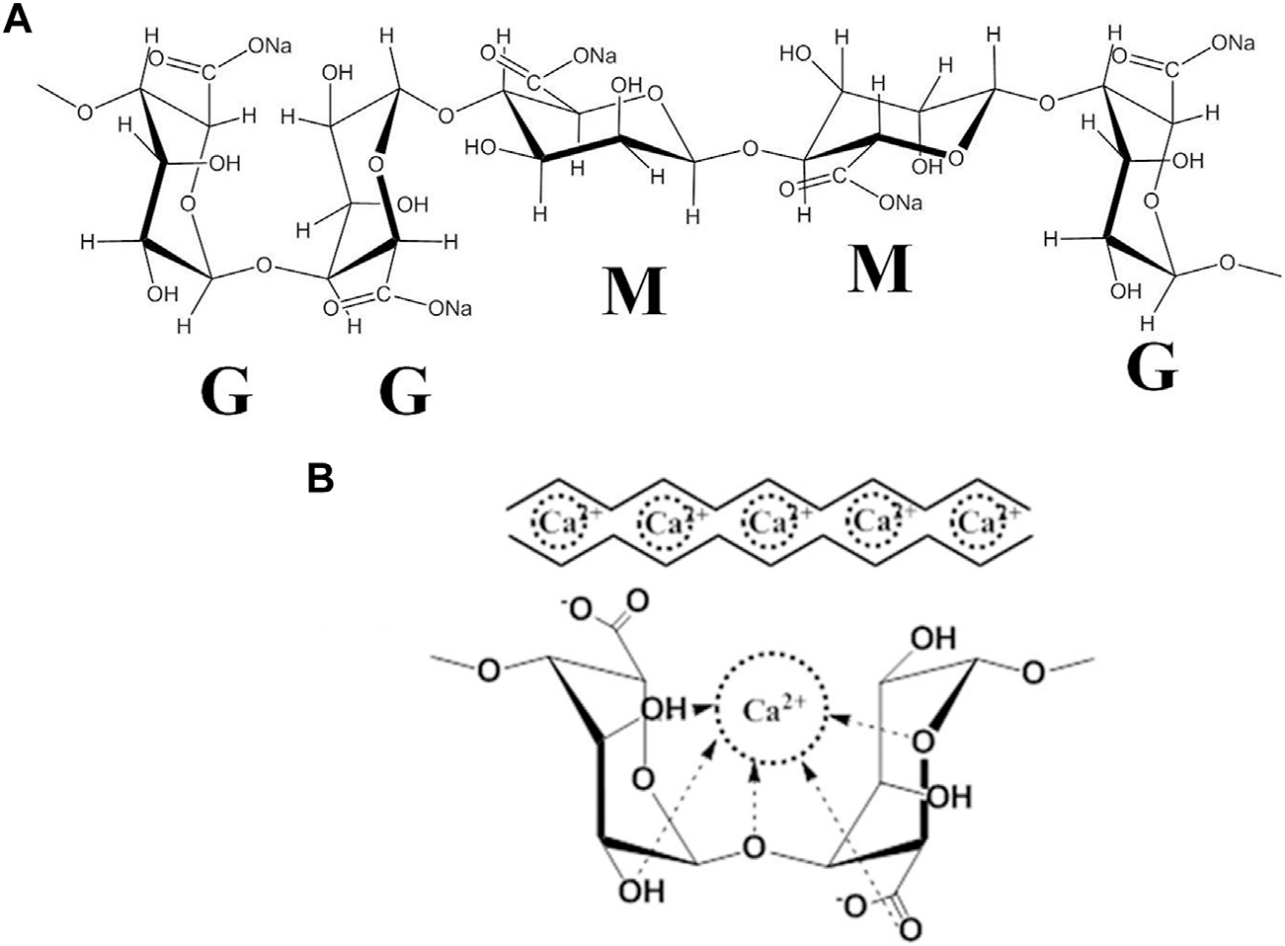
**(A)** The structure and block distribution of sodium alginate. **(B)** The “egg-box”model is formed by the cross-linking of the G-unit of alginate with Ca^2+^.

Cartilage tissue engineering provides a promising strategy for regenerating damaged articular cartilage. The extracellular matrix (ECM) supports the chondrocytes during culture, maintaining their characteristics or recovering their phenotypes (Shi et al., 2017; Zhang et al., 2020). The effects of cell-matrix interactions on chondrogenesis and cartilage tissue development have not yet been clearly articulated. Therefore, finding a suitable matrix component that can maintain the viability, phenotype stability, and functionality of chondrocytes is crucial in cartilage regeneration. Owing to similarities in their structural and mechanical stiffness, hydrogels with a 3D porous structure have exhibited a great advantage in serving as *in vivo* microenvironments that mimic the natural cartilaginous ECM for chondrocyte growth (Lin et al., 2020). Moreover, several studies have proved that the cell viability and maintenance of the cellular phenotype can be altered by the hydrogel matrix mechanical properties (An et al., 2018). Alginate (ALG) is a well-known natural polysaccharide composed of *a*-L-guluronate (G-units) and *ß*-D-mannuronate acid (M-units) (Figure 1A). Particularly, the M-units mainly lead to alginate molecules showing a “linear” arrangement, while an abundance of G-units confers to the alginate molecules an irregular “zig-zag” structure (Dodero et al., 2021). More importantly, the G-units can easily undergo ionotropic gelation with divalent cations (such as calcium or magnesium ions) to form the “egg-box” structure under physiological conditions (Figure 1B). Specifically, the ionically cross-linked alginate hydrogel can be applied to the desired body site without using potentially toxic or costly chemical cross-linking reagents to deliver cells and bioactive molecules, which have been widely used in cartilage tissue engineering, showing encouraging results (Farokhi et al., 2020). For example, it has been shown that the alginate-based scaffold significantly enhances chondrocyte cell proliferation. It has also been shown to support the redifferentiation potential of dedifferentiated chondrocytes (Lee et al., 2017).

However, despite these advantages, the applications of alginate are greatly limited by drawbacks such as the lack of cell adhesion anchoring, resulting in functional disfavor (Mahou et al., 2018). To make up for this drawback, bioactive molecules were introduced into alginate to provide biochemical functionality for cell growth (Gentile et al., 2017). Collagen (COL) is the major part of the cartilage ECM, which can present essential binding motifs to promote cell adhesion and cell proliferation. It is highly recommended for synergy with other biopolymers in cartilage tissue engineering to prepare hydrogels for cell stimulation. In fact, some studies have reported the beneficial role of COL in preventing cell dedifferentiation (Yuan et al., 2016; Cai et al., 2019). Thus, COL was introduced into the ALG hydrogel matrix in our study. On the other hand, the cell-cell interaction of a scaffold is crucial in tissue engineering. COL, particularly in relation to ALG molecules, induces a possible chemical interaction whereby the amine group of COL can interact with the carboxyl group of ALG (Mahapatra et al., 2016), resulting in improved hydrogel stability and enhanced cell-cell and cell-matrix interactions. Therefore, we propose to use the interpenetrating network hydrogel prepared by sodium alginate and collagen as the chondrocyte culture system, which not only ensures the presence of sufficient adhesion sites that are conducive to cell adhesion and proliferation through collagen molecules but also regulates the mechanical properties of the hydrogel through the introduction of sodium molecules.

The matrix stiffness and elastic modulus of the hydrogel have critical effects on the synthesis of the extracellular matrix of chondrocytes and the maintenance of chondrocyte phenotype and function. Studies have shown that the morphology and cytoskeletal organization of chondrocytes can be altered by tuning the mechanical properties of the hydrogel matrix (Nicodemus et al., 2011; Lee et al., 2017; Lou et al., 2017; Ma et al., 2018; Seo et al., 2019). However, recent studies have mostly focused on the effect of the molecular weight (M_*w*_) of alginate on chondrocyte activity by regulating mechanical strength (Nicodemus et al., 2011; Lou et al., 2017; Ma et al., 2018). To date, the effect of the alginate uronic acid composition on the biological response of such systems remains somewhat unclear. The G/M ratio is another crucial physical property of sodium alginate, which can also affect the strength of the alginate gel. G-rich alginates are more prone to ionic gelation and can provide higher strength and stability in the final hydrogel product (Kakita and Kamishima, 2008; Fu et al., 2011; Liu et al., 2020). Thus, it is essential to investigate the effect of the G/M ratio and the chemical properties of alginate on the morphology and function of chondrocytes when designing chondrocyte cell culture systems related to alginate hydrogels.

In this work, we developed an interpenetrating polymer network hybrid hydrogel composed of ALG and COL to render the resulting ALG/COL hydrogel suitable for seeding with chondrocytes. Based on previous studies, we hypothesized that the G/M ratio of alginate used to prepare ALG/COL hydrogels may alter the microenvironment for the culture, maintaining and expanding chondrocytes without losing their phonotype. To demonstrate the hypothesis, ALG/COL hydrogels with a high G/M ratio and mid G/M ratios were fabricated and characterized. In addition, the effects of the G/M ratio of alginate on chondrocyte viability, morphology and expression levels of cartilage-specific genes were evaluated *in vitro*. Finally, the effects of G/M ratios of alginate on the mechanical stiffness and surface properties of the ALG/COL hydrogel will be investigated subsequently to explain the phenotype and functionalization of the cultured chondrocytes. We believe that this study will provide new insights into the design of potential alginate-based composites for cartilage tissue engineering.

## 2 Materials and methods

### 2.1 Materials

Sodium alginate with molecular weight (M_*w*_) of 520 kDa and G/M ratio of 64/36 was designated High-G/M alginate, and sodium alginate with M_*w*_ of 532 kDa and G/M ratio of 34/66 was designated Mid-G/M alginate. Both were purchased from Qingdao Mingyue Seaweed Group Co., Ltd. (Qingdao, China). The detailed information of both purchased alginates was characterized by GPC and ^1^HNMR spectroscopy (Table 1).

**TABLE 1:** Chemical composition, sequence parameters and molecular weight of the alginates used; *F*_*M*_, *F*_*G*_ is the fraction of mannuronic (M) and guluronic (G) acid, respectively. The composition and segment frequencies of two mannuronic (MM), guluronic (GG) or mixed (MG) acids were determined by ^1^HNMR. The mass average molecular weight (M_*w*_) was determined by GPC.

*Alginate*	*F*_G_	*F*_M_	*F*_GG_	*F*_MG_	*F*_MM_	*M*_*W*_ *(kDa)*	*Mw/M*_*n*_
*Mid-G/M*	0.34	0.66	0.17	0.16	0.50	532	1.32
*High-G/M*	0.64	0.36	0.54	0.10	0.26	520	1.29

The G/M ratio of the respective alginates was determined at 80°C by ^1^HNMR spectroscopy using an AVANCE NB-360 spectrometer (Bruker, Germany). The M_*w*_ and molecular weight distribution (M_*w*_/M_*n*_) of alginate samples were analyzed *via* gel permeation chromatography (GPC) using a system equipped with two TSK-gel size-exclusion columns (G4000PWXL and G3000PWXL), a refractive index detector (Waters, model 2414, Milford, MA), and an HPLC pump (Waters, model 515).

Collagen, acquired from rat tails, and was obtained by our laboratory. Anhydrous calcium chloride (CaCl_2_) was purchased from Sigma-Aldrich (Merck Life Science (Shanghai) Co., Ltd.). All other chemical reagents were analytical grade and used as received.

### 2.2 Fabrication of alginate/collagen (ALG/COL) hybrid hydrogels

The hydrogel was prepared according to the method previously described with some modifications (Song et al., 2016). Briefiy, sodium alginates (High-G/M and Mid-G/M) were dissolved in 0.9% (w/v) NaCl solution to a final concentration of 60 mg/mL. Collagen solution (COL) (6 mg/mL dissolved in 0.02 M acetic acid) was neutralized to a pH of seven by adding 1 M NaOH aqueous solution. The resulting alginate and collagen solutions were mixed at a 1:2 (V/V) ratio, respectively. And then homogenized by magnetic stirring for 4 h at 4°C. The obtained homogeneous ALG/COL blend was finally degassed by GT16-3 tabletop refrigerated centrifuge at 200 g for 10 min 4°C, and 0.6 mL of the resulting solution was gently dropped onto circular glass coverslips (diameter = 3.0 cm). After gelation at 37°C for 2 h, the sample was further immersed in CaCl_2_ solution (1.1%) for 30 min to form the ALG/COL hybrid hydrogel.

### 2.3 Characterizations of physicochemical properties

The microstructure and morphology of the two types of samples were observed with a scanning electron microscope (SEM; JSM-6360 LV). To obtain the measurements, the samples were gradient dehydrated with ethanol. The dehydrated hydrogel was then further dried with a critical point dryer and sputter-coated with gold, mounted on aluminum stubs, and examined with a SEM using an accelerating voltage of 20 kV.

The chemical bond structure of the samples was analyzed *via* attenuated total reflectance Fourier transform infrared (ATR-FTIR) spectroscopy (Nicolet iS50, Thermo Scientific) equipped with a Smart Endurance diamond ATR accessory (32 scans, resolution of 4 cm^-1^, and wavenumber range of 4,000–400 cm^-1^).

The surface roughness of hydrogel samples was analyzed using an optical interferometer (New-View 5020, ZYGO) in a wet state. Average values were acquired from multiple roughness values (at least three) at random regions of each sample.

### 2.4 Mechanical measurements

The compressive modulus of hybrid hydrogels was carried out by a universal testing machine (Instron 3342; Norwood, MA, United States). Cylindrical samples with a diameter of 10 mm and a height of 5 mm were placed between the bottom and upper plates of the machine for texting. The load cell is a force transducer with a range of 0–50 N (measuring accuracy of 1 mN). The compression rate was 0.1 mm/min and the samples were tested in wet state. The compression test was performed after the related parameters were set. Experiments were terminated by compression to 50% strain of the initial height and all experiments were performed at the room temperature. The compressive modulus was calculated from the initial linear regions (strains from 5% to 30%) of the stress-strain curves. The compressive test for each sample consisted of five replicates for statistical analysis.

### 2.5 Isolation and culture of chondrocyte

Primary chondrocytes were obtained from the cartilage tissues of 1-month-old New Zealand white rabbits bought from the Medical Animal Experimental Center of Dalian Medical University (Dalian, China). The animal experimental procedure was approved by the Institutional Animal Ethics Committee of the Affiliated Zhongshan Hospital of Dalian University. An established protocol was described elsewhere (Shimomura et al., 1975). Briefly, the cartilage tissues were cut into a small cube and digested with trypsin (Solarbio, China) for 30 min at 37°C. After being washed three times with Dulbecco’s modified Eagle medium (DMEM; Gibco, United States), the cartilage was placed into DMEM containing type II collagenase (2 mg/mL; Gibco) and incubated at 37°C for 3 h under shaking conditions. The digested tissues were centrifuged at 150 g for 5 min and then filtered through a sterile 200-mesh strainer. After centrifugation, the chondrocytes were resuspended and cultured in chondrocyte culture medium (DMEM/F12; Gibco) supplemented with 10% fetal bovine serum (FBS; Gibco) and 1% antibiotic solution (10 mg/mL streptomycin and 100 U/mL penicillin). At passage 3, when 80%–90% fusion occurred, chondrocytes were used for the *in vitro* cell test.

### 2.6 Cell culture on ALG/COL hydrogel for the in vitro test

The ALG/COL composite hydrogels were prepared according to the method described above. Chondrocytes were resuspended in the culture medium to achieve a concentration of 1 × 10^5^ cells/mL. After loading the 600 μL hydrogel into a 24-well plate, 0.5 mL volume of the cell suspension was added to the corresponding membrane and placed in an incubator.

### 2.7 Cell viability and cell proliferation

Cell viability was evaluated using a Live/Dead staining assay kit (Invitrogen, United States) based on a method described previously (Zheng et al., 2016). Cell/hydrogel constructs were stained with calcein AM (2 μM) and ethidium homodimer-1 (EthD-1, 4 μM) for 30 min and then observed under a confocal laser scanning microscope (CLSM; Leica SP2, and Germany).

The CCK-8 assay was used to assess cell proliferation. Chondrocytes cultured on ALG/COL composite hydrogels with different G/M ratios were placed in a 24-well plate for 1, 4, and 7 days at a density of 10,000 cells per well. At each time point, the cell-hydrogel constructs were incubated in a medium containing 10% CCK-8 solution at 37°C for 2 h, the absorbance was measured at 450 nm. The tests were performed in triplicate.

### 2.8 Observation of cell adhesion by SEM

Cell adhesion on both hydrogel surfaces was observed *via* SEM (JSM-6360 LV) with an accelerating voltage of 20 kV. After incubation for 7 days, the samples were fixed in 2.5% glutaraldehyde at 4°C overnight. After washing with phosphate-buffered saline (PBS), the samples were dehydrated with a graded ethanol series for 25 min at each gradient, dried in hexamethyldisilazane (HMDS), coated with platinum, and evaluated *via* SEM.

### 2.9 Cytoskeleton staining

To detect actin filaments, after 7 days of incubation, chondrocytes on ALG/COL composite hydrogels with different G/M ratios were gently rinsed three times with PBS and fixed with paraformaldehyde (4%). Subsequently, the sample (n = 3) was incubated with 0.5% Triton X-100 in PBS for permeability. After washing with PBS, the cells were stained with 1 μg/mL rhodamine/phalloidin (Invitrogen, United States) for 30 min at room temperature in darkness. Nuclei were stained with 10 μg/mL DAPI solution (Beyotime, United States), and images were obtained using a CLSM.

### 2.10 Real-time PCR analysis

Gene expression of collagen type II (COL2), SOX9, aggrecan, Ras-related C3 botulinum toxin substrate (RAC), and Rho-associated coiled coil containing protein kinase 1 (ROCK1) were determined using real-time qRT-PCR to assess chondrocyte responses to altered G/M ratios. The chondrocytes on both hydrogels were harvested for RT-PCR at seven- and 14-day time points. At harvest, the cell-scaffold constructs were washed three times by PBS. Subsequently, 0.055 mol/L sodium citrate was added to the constructs to dissolve the alginate for 15 min and was washed twice with PBS. After which, the samples were incubated with 2 mL of 0.3 mg/mL collagenase for 10 min at 37°C, followed by stopping digestion with serum-containing medium. Finally, the cells were centrifuged at 500 g for 15 min and the supernatant was discarded to obtain chondrocytes. The RNAiso Plus (Takara, Tokyo, Japan) was used to isolate total RNA. cDNA was obtained using a PrimeScriptTM RT-PCR Kit (Takara, Tokyo, Japan). Quantitative real-time PCR was performed to determine gene expression using an ABI Prism 7,300 System (Applied Biosystems, United States). The RT-PCR amplification process was performed under the conditions of denaturation at 95°C for 15°min, followed by 40 cycles consisting of 94°C for 15°s and 60°C for 34 s. The relative transcript quantities were calculated according to the 2^−ΔΔCt^ method. The glass coverslip group (without hydrogel) was considered as the untreated sample in the delta CT calculations. Glyceraldehyde 3-phosphate dehydrogenase (GAPDH) was set as the control gene. In this experiment, each group and sample test had been performed in triplicate. The forward and reverse primer sequences of the genes used for RT-PCR are listed in Table 2.

**TABLE 2:** Primer sequence used *in vitro* experiments.

Gene name		Sequences
Aggrecan	Forward	CTGCCTCAGGGATCCGTAAAG
	Reverse	CCTCTGCCTCAGGAATGACAT
SOX9	Forward	CCTTGAGTCCTTGCGCGGCA
	Reverse	TTGGCCCTCCTCCTCCAGCC
Collagen II	Forward	CACCACGCTCTTCTGTCTACTGAAC
	Reverse	TGCCACAAGCAGGAATGAG
GAPDH	Forward	GACTGATGTTGTTGACAGCCACTGC
	Reverse	TAGCCACTCCTTCTGTGACTCTAAC
RAC	Forward	GAGAGTACATCCCCACCGTC
	Reverse	AACACGTCTGTTTGCGGGTA
ROCK1	Forward	TTGGTTGGGACGTACAGTAAAA
	Reverse	CGTAAGGAAGGCACAAATGAGA

### 2.11 Quantitative analysis of extracellular matrix

The contents of glycosaminoglycan (GAG) and collagen II in the ECM secreted by chondrocytes were quantified. Samples (n = 5) were rinsed in PBS, cut, and homogenized in PBS with a glass grinder. The resulting suspension was subjected to two freeze-thaw cycles to break the cell membrane further. The supernatant was immediately removed by centrifugation at 500 g value for 15 min, after which the supernatant was immediately removed. The contents of GAG and collagen type II were measured using a rabbit GAG enzyme-linked immune sorbent assay (ELISA) kit and a collagen type II ELISA kit (Blue Gene Biotech, Shanghai) according to protocols provided by the manufacturer. Five samples were used for each biochemical analysis.

### 2.12 Statistical analysis

In this study, all experiments were repeated at least in triplicate. The representative data were manifested by mean ± standard deviation (SD). Statistical analysis was performed with Student’s *t*-test and a one-way analysis of variance (ANOVA) to analyze the differences between groups. A *p*-value of <0.05 was accepted as statistical significance (*p < 0.05, **p < 0.01, ***p < 0.001).

## 3 Results and discussion

### 3.1 Formation and characterization of ALG/COL hydrogel

**FIGURE 2:**
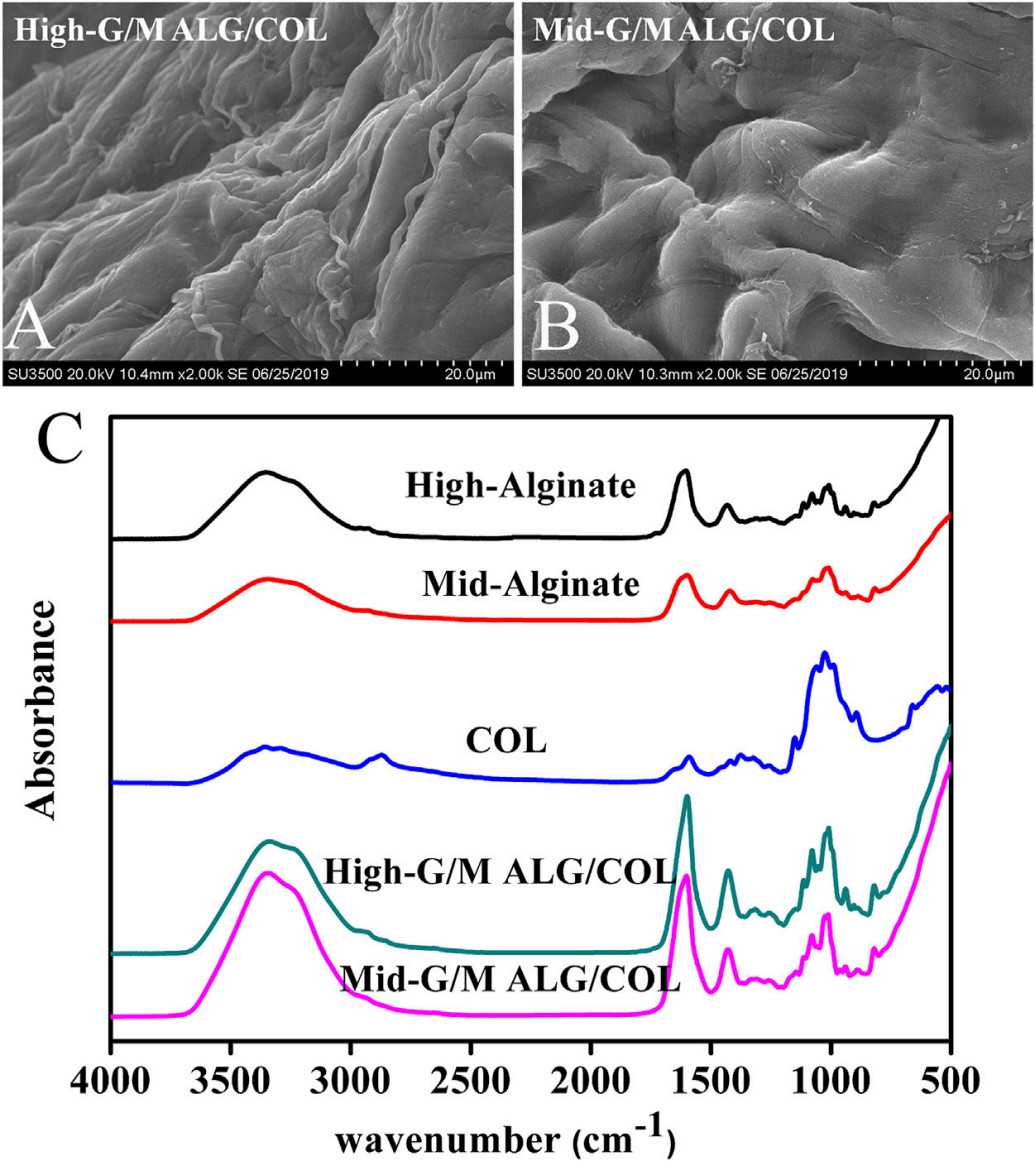
SEM images of the morphology of **(A)** High-G/M and **(B)** Mid-G/M ALG/COL hydrogels. **(C)** FTIR spectra of High-G/M and Mid-G/M ALG/COL hydrogels.

The morphology of hydrogels has an important influence on cell behavior and matrix expression (Dong et al., 2021; Liu et al., 2022). In the present study, the microstructural morphology of the hydrogels was first investigated *via* SEM, as shown in Figure 2. Mid-G/M ALG/COL hydrogels exhibited a substantially loose and porous framework (Figure 2B), whereas High-G/M ALG/COL hydrogels demonstrated a much denser and less porous morphology (Figure 2A). This is probably due to the formation of alginate hydrogels by the establishment of ionic bridges between Ca^2+^ and G blocks in the alginate chain. High-G/M alginate provided sufficient G blocks for Ca^2+^ ions to be fully bound, allowing a dense organization of the hydrogel structure, improving structural integrity, and creating less porosity.

Furthermore, the possible chemical interactions between the components and the structural properties of the hydrogel were evaluated *via* ATR-FTIR. The ATR-FTIR spectra of High-G/M alginate, Mid-G/M alginate, COL, Mid-G/M ALG/COL, and High-G/M ALG/COL hydrogels are provided in Figure 2C. The absorption peaks of sodium alginate at approximately 3,434 cm^−1^ and 1,600 cm^−1^ were attributed to the -OH groups and the stretching vibration of the carboxyl group, respectively. The characteristic absorption peaks can be seen in samples of ALG/COL network hydrogels, indicating the presence of alginate molecules in the composite hydrogels. In another aspect, pure COL has characteristic amide peaks (amide I at ∼1,650 cm^-1^ and amide II at ∼1,400 cm^-1^), which are directly related to the *a* helix, *ß* folding, and irregular curl structure of its secondary structure (Chen et al., 2019). For both types of hydrogel, strong amide I and II peaks were found in the composite hydrogels, suggesting that COL had been successfully introduced into the hydrogels. Therefore, it can be seen that no molecular interaction had occurred between alginate and collagen in the two types of hydrogels, which indicated the ALG/COL hybrid hydrogels were interpenetrating network (IPN) hydrogels prepared by ionotropic gelation of alginate combined with collagen fibrils. Similar alginate-based IPN (an acronym for interpenetrating network) hydrogels have previously been reported (da Cunha et al., 2014; Mahou et al., 2018). In this study, the ALG/COL hybrid hydrogels were further confirmed as IPN structures from the ATR-FTIR spectra.

### 3.2 Effect of hydrogels with different G/M ratios on chondrocyte viability and proliferation

**FIGURE 3:**
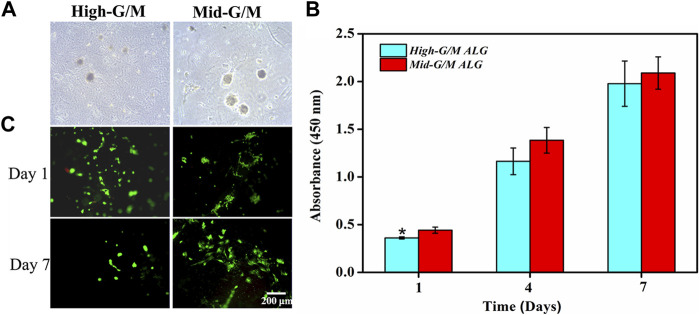
Proliferation and live/dead staining of chondrocytes on ALG/COL hybrid hydrogels. **(A)** Phase contrast images of chondrocytes cultured on hydrogels with different G/M ratios for 3 days. **(B)** Cell proliferation was determined by CCK-8 assay for 1, 4, and 7 days. **(C)** Images of live and dead chondrocytes cultured on ALG/COL hydrogels with different G/M ratios. Dates are represented as the mean ± SD (n = 3). (*p < 0.05 indicates a significant difference between the experiments).

Both types of hydrogels were seeded with chondrocytes, and the morphology of cells was observed using phase contrast microscopy (Figure 3A). The cells were present at low density with some cells exhibiting cellular spherical aggregates after a few days of culture (typical cell shape of native chondrocytes). In particular, larger and more numerous cell aggregates were observed on the surface of the Mid-G/M sample compared with those on the surface of the High-G/M sample. Chondrocytes can develop spherical aggregates through self-assembled interactions with neighboring cells. These aggregates play a vital role in cartilage regeneration because cell aggregation normally indicates beneficial chondrogenic differentiation (Chen et al., 2016). In addition, CCK-8 assay was used to assess chondrocyte proliferation on both hydrogels. The data revealed that cells proliferated well on both hydrogels, and there was no significant difference in the proliferation ability of chondrocytes between the two groups during the 7 days of culture time (*p* = 0.5472) (Figure 3B). Moreover, live/dead assay showed that the majority of cells on both hydrogels were stained green, indicating good viability of the cells (Figure 3C). Therefore, the above data suggest that ALG/COL composite hydrogels can be considered to provide matrix conditions suitable for chondrocyte proliferation, and that the Mid-G/M sample group may be more favorable for maintaining the chondrocyte phenotype required for cell therapy in cartilage engineering.

### 3.3 Effects of hydrogels with different G/M ratios on cytoskeleton morphology

**FIGURE 4:**
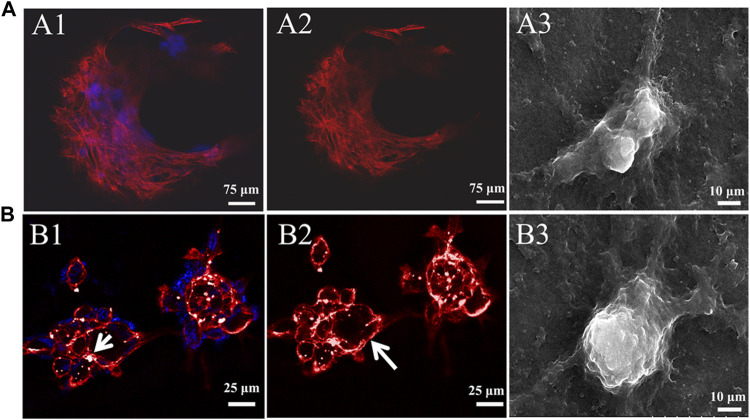
CLSM images of the cytoskeleton of chondrocytes on **(A)** High-G/M (A1-A2) and **(B)** Mid-G/M (B1-B2) ALG/COL hydrogels after 7 days of culture *in vitro*. Cytoskeleton (red), nucleus (blue). Scale bars are 25 μm. A white arrow indicates a rounded cell aggregate. SEM micrographs of chondrocytes cultured on composite alginate hydrogels with (A3) High-G/M and (B3) Mid-G/M samples, respectively. (5000 **×** magnification, scale bar = 10 μm).

Cell adhesion to the ECM is vital for cell survival, proliferation, and the expression of phenotype differentiation (Danen and Sonnenberg, 2003). Cytoskeletal morphology was observed using rhodamine-phalloidin labeling of the actin filaments to explore cytoarchitecture changes in chondrocyte responses to High-G/M and Mid-G/M ALG/COL hydrogels (Figure 4). Drastic alterations in F-actin distribution were observed after 7 days of incubation. The chondrocytes cultured on High-G/M ALG/COL hydrogels displayed wide spreads and anomalous shapes with prominent, deeply organized paralleled actin fibers (Figure 4A). While chondrocytes cultured on the Mid-G/M ALG/COL groups, yielded a round and more stereoscopic globular shape with disorganized actin filaments localized at the periphery of the chondrocytes (Figure 4B). Moreover, the cell aggregates were larger on the Mid-G/M ALG/COL hydrogels than on the High-G/M hydrogels. Previous studies have shown that cell-cell interactions are important during cartilage formation, and the volumetric structure of cell aggregates enhances chondrogenesis *via* extremely tailored cell-cell interactions (Bush and Hall, 2003; Ling et al., 2021). Thus, we hypothesized that the Mid-G/M ALG/COL hydrogel could induce stronger cell-cell interactions, resulting in the formation of a condensed structure (Liu et al., 2022). Therefore, the finding implied that the Mid-G/M ALG/COL hydrogels may provide a matrix condition favorable for chondrocytes to multiply in number and maintain phenotype, which is crucial for sustaining normal chondrocyte functionalization. This result also corresponds to our observations *via* SEM (Figures 4A3, 4B3). And other areas of the samples taken by SEM also showed the same results (data not shown in the main text).

### 3.4 Cartilage specific gene expressions and cartilage matrix formation

**FIGURE 5:**
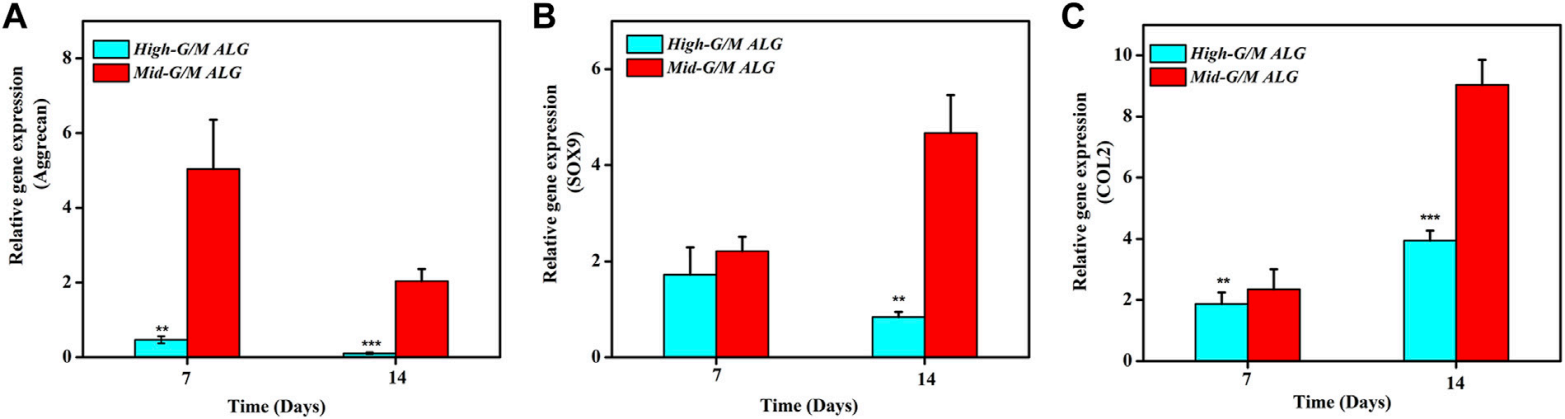
Effects of High-G/M ALG/COL and Mid-G/M ALG/COL hydrogels on mRNA expression, as quantified by 2^−ΔΔCt^ at 7 and 14 days. **(A–C)**: qPCR analysis of Aggrecan, SOX9 and COL2 mRNA expression. Dates are represented as the mean ± SD (n = 3). (**p < 0.01, ***p < 0.001 indicate significant differences among the experiments.

**FIGURE 6:**
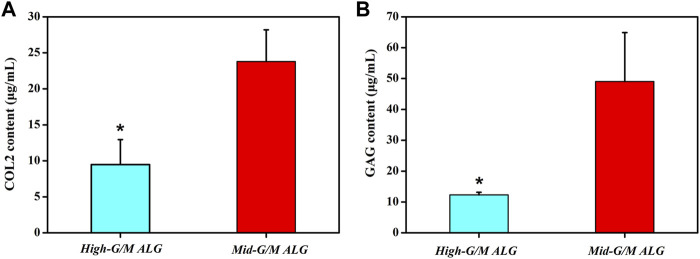
Quantification of **(A)** collagen type II (COL2) and **(B)** matrix production (GAG) of chondrocytes on High-G/M ALG/COL and Mid-G/M ALG/COL hydrogels evaluated *via* ELISA. Dates are represented as the mean ± SD (n = 3). (*p < 0.05 indicates a significant difference in the number of experiments).

The effect of the G/M ratios on chondrogenic differentiation was subsequently studied by characterizing cartilage-specific markers. The gene expression of cartilage-specific markers with High-G/M and Mid-G/M ALG/COL hydrogels was analyzed using real-time PCR analysis. Collagen type II (COL2) is a special component in hyaline cartilage and has been recognized in cartilage development. SOX9, an essential gene in the formation of cartilage, directly promotes the synthesis of COL2. Proteoglycans, as a primary component of the extracellular matrix, play a major role in sustaining the load-bearing capacity of cartilage (Egunsola et al., 2017). The expression levels of the aggrecan (*p* = 0.0039 and 0.0005, respectively), SOX9 (*p* = 0.2579 and 0.0011, respectively), and COL2 genes (*p* = 0.0072 and 0.0006, respectively), which are cartilage-specific genes, were significantly higher when cultured on the Mid-G/M than on the High-G/M hydrogels after 7 and 14 days of culture (Figures 5A–C). This indicates that the Mid-G/M ALG/COL hydrogels have a greater potential to enhance chondrocyte differentiation than the High-G/M group. Moreover, the COL2 and GAG contents were significantly enhanced by Mid-G/M ALG/COL than by High-G/M ALG/COL, as demonstrated by the quantification analyses *via* ELISA (*p* = 0.0114 and 0.0161, respectively, Figure 6). The results demonstrated that chondrocytes cultured on Mid-G/M hydrogels presented a high capability to produce major ECM molecules in cartilage, suggesting that Mid-G/M ALG/COL gels could provide a more suitable biophysical (mechanical and stability characteristics) and biochemical (adhesion motifs) environment for chondrocytes to develop a cartilage-like matrix during long-term *in vitro* culture. Thus, the Mid-G/M ALG/COL hydrogel should be an ideal biomaterial to mimic natural ECMs.

### 3.5 Effects of hydrogels with different G/M ratios on the mechanical properties of ALG/COL hydrogels

**FIGURE 7:**
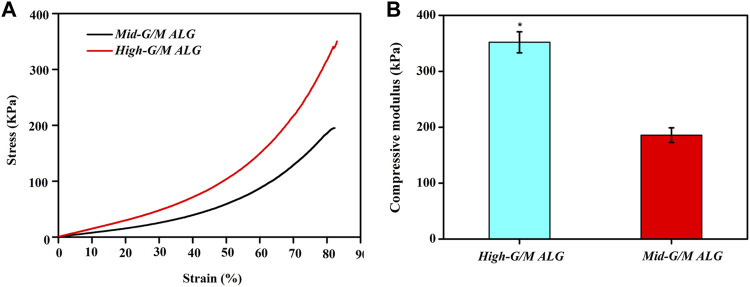
Compressive stress-strain curves **(A)** and compressive modulus **(B)** of ALG/COL hydrogels. The compressive modulus is calculated from the slope of the initial linear portion of the stress-strain curve. Results are expressed as mean ± standard deviation (SD). (*p < 0.05 indicates a significant difference between the two groups).

**FIGURE 8:**
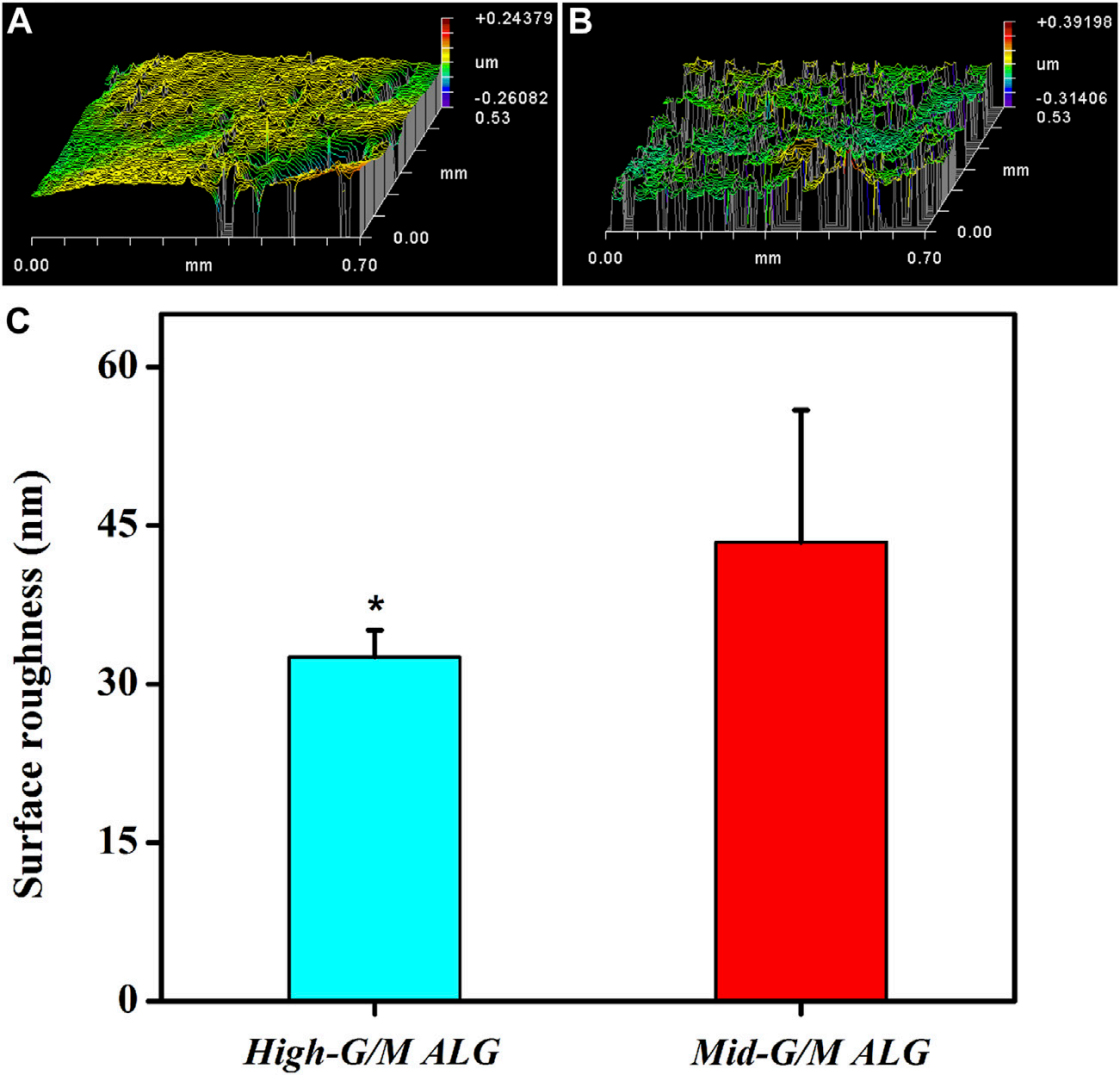
Representative images of the surface morphology of both hydrogels in a wet state: **(A)** High-G/M ALG/COL and **(B)** Mid-G/M ALG/COL. **(C)** Quantitative analysis of the surface roughness of the two types of hydrogels (*p < 0.05 indicates a significant difference in the number of experiments).

The above findings clearly indicate that the phenotype and function of chondrocytes during *in vitro* culture are significantly different for both types of hydrogels. These findings motivate further exploration of the effect of the G/M ratio of the alginate used to prepare the hydrogels on the physicochemical properties of the resulting ALG/COL composite hydrogels and the consequent signals transmitted into the chondrocytes, allowing them to adjust to the surrounding microenvironment. It is well known that the ECM environment of chondrocytes plays an essential role in determining the regenerative quality for cartilage tissue engineering. Besides, previous studies proved that the substrate stiffness should be highly focused on for better chondrocyte functionalization (Schuh et al., 2010). Chondrocytes can perceive and respond to the mechanical properties of the ECM (Zhao et al., 2020). Therefore, the influence of the G/M ratios on the mechanical properties of ALG/COL hydrogel was first analyzed by the universal Testing Machine measurement (Figures 7A, B). ALG/COL composite hydrogels with different G/M ratios showed different compressive moduli. The modulus of both groups were in the range of ∼80–350 kPa, and the High-G/M hydrogel group revealed a significantly higher value (350 kPa) than the Mid-G/M group (180 kPa) (*p* < 0.05, Figure 7B). The results showed that alginate with a higher G/M ratio enhanced the mechanical properties of the hydrogel. The ALG/COL hydrogel is composed of two main network structures: the polymer network (the physical entanglement of polymer chain segments between sodium alginate and collagen) and the ion network (the interaction between sodium alginate and calcium ions). The stiffness of the composite hydrogel with a high G/M ratio increased mainly due to the electrostatic attraction between the alginate and collagen chain segments and the alginate-Ca interaction. The use of High-G/M ALG/COL hydrogels may lead to significantly enhanced alginate-Ca interactions in the hydrogels. Moreover, the active functional group (amine) of the collagen may conjugate and entangle within the ALG backbone because of its inherent rigidity, the High-G/M ratio alginate being in an aligned stable backbone due to the represented rod-like shape, which may induce stronger physical entanglement and hydrophobic interactions between alginate and collagen and consequently reinforce the stiffness of High-G/M ALG/COL hydrogels (Chen et al., 2019). Thus, the stiffness of ALG/COL hydrogels can typically be regulated by varying the G/M ratio of the polymer. Indeed, it has been shown that the G/M ratio of the alginate plays a key role in determining the stiffness of the hydrogel. For example, Costa et al. (2018) reported that the mechanical properties of alginate-based hydrogel films should take into account the G/M ratios since high contents of G residues contribute to an increase in mechanical properties. Gómez-Mascaraque et al. also verified that higher G/M ratios lead to higher mechanical strength than lower G/M ratios (Gómez-Mascaraque et al., 2019). This could also be attributed to the polymeric chains of alginate with a higher G/M ratio leading to higher cross-links in the hydrogel network, resulting in a stronger hydrogel. Moreover, other studies reported on the stiffness of the pericellular matrix of human cartilage, illustrating that stiffness ranging from 17 to 200 kPa is the most suitable for chondrocytes (Kourouklis et al., 2016; Tao et al., 2016). In yet another study, the cartilage ECM production (Collagen type II and GAGs) was enhanced when chondrocytes were cultured on gels with a stiffness of approximately 190 kPa (Thomas et al., 2017). Hence, a Mid-G/M ALG/COL hydrogel with a stiffness of 180 kPa should more closely mimic the stiffness of the native cartilage environment, which is beneficial for chondrocyte sensing and cartilaginous matrix generation. In addition to stiffness, other physical properties, such as topography, also have an effect on the changes of its recovering cells. Therefore, we characterized the surface roughness of both samples with a white-light profilometer. The results showed that High-G/M and Mid- G/M ALG/COL hydrogels had roughness values of 32.56 nm and 43.41 nm (Figure 8), respectively. Statistical differences were observed between every two samples (*p* < 0.05). However, previous studies proved that the substrates with surface roughness below 45 nm had a slight effect on cell behavior (Zhao et al., 2020) and that there was no difference in cell mechanosensitivity when cultured on substrates with nanotopography <20 nm in height (Salvi et al., 2010; Hu et al., 2011). Therefore, surface roughness does not seem to be a crucial factor in this outcome.

**FIGURE 9:**
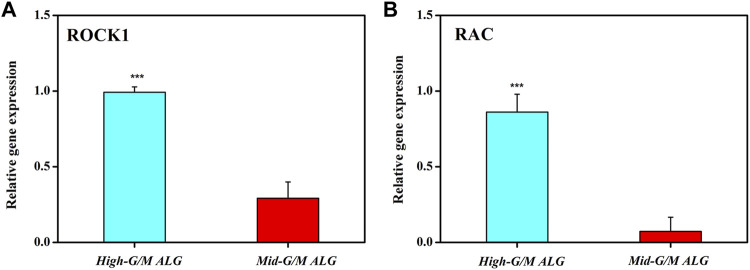
Effects of the G/M ratios of ALG/COL hydrogels on **(A)** ROCK1 and **(B)** RAC mRNA expression in chondrocytes, as quantified using the 2^−ΔΔCt^ method at 14 days. Dates are represented as the mean ± SD (n = 3). (***p < 0.001 indicates a significant difference between the experiments).

Moreover, we found that the stiffness of the ALG/COL hydrogel plays an important role in chondrogenic differentiation. Previously, it was reported that the suitable mechanical properties of hydrogels are essential for chondrocyte differentiation and the development of cartilage tissue (Yuan et al., 2016). In addition, the matrix stiffness has recently been highlighted as the key role in controlling chondrocyte behavior (cell adhesion and cytoskeleton morphology) and cell-produced ECM activity (Wang et al., 2014). Therefore, to point out the detailed mechanical conduction effect between the aforementioned chondrocytes and the extracellular matrix, which support the cell phenotype (chondrogenesis) and ECM production, the underlying signaling pathway associated with the cell-matrix mechanotransduction process (RhoA/ROCK signaling suppressed chondrogenesis) was further investigated (Ridley, 2000). The expression of ROCK1 and RAC genes, which has significantly been correlated with the regulation of actin cytoskeleton as well as actomyosin contractility, was examined (Figure 9). Chondrocytes were found to display significantly higher expression levels of both ROCK1 and RAC on hydrogels with High-G/M after 14 days of culture (*p* < 0.001), consistent with the group that had lower expression of COL2 and aggrecan, which indicates that High-G/M ALG/COL may suppress chondrogenesis. A previous study demonstrated the existence of a dynamical equilibrium between the traction force exerted by the cell and the force exerted by the extracellular matrix during the mechanosensing process. On soft substrates, cell morphology is directly affected by the stiffness of the material. In general, cells contract their actin fibers to exert force on the ECM. When the resistance of the cell equals the applied force by the deformation of the ECM, a stable morphology will be maintained (Chen et al., 2016). Together with previous literature on the relationship between maintaining chondrocyte phenotype and reconstruction of the cytoskeleton (Woods et al., 2005), our study demonstrated that Mid-G/M ALG/COL composite hydrogels with a mechanical stiffness of 180 kPa seemed to be more suitable for use as substrates for chondrocyte culture, facilitated more collagen expression, and supported chondrocyte phenotype maintenance over long timescales, indicating that the stiffness of the hydrogel substrate should be properly tuned to match the native ECM elasticity while designing an *in vitro* microenvironment for cartilage regeneration.

## 4 Conclusion

An alginate/collagen (ALG/COL) hybrid hydrogel chondrocyte model culture system was developed using different G/M ratios of alginate and collagen *via* a physical cell-friendly gelation method. The G/M ratios of alginate used to prepare ALG/COL hydrogels could influence the stiffness of the resultant ALG/COL composite hydrogels and further influence the chondrocyte behaviors. Larger and more cell aggregates can be found when chondrocytes were cultured on the surface of ALG/COL hydrogels with Mid-G/M ratios. Moreover, cartilage-specific genes, namely, aggrecan, SOX9, and COL2, were significantly more highly expressed, but the RAC and ROCK1 genes were downregulated in the Mid-G/M ALG/COL composite hydrogel group. In addition, Mid-G/M ALG/COL hydrogels may improve the chondrocyte phenotype and enhance functionalization due to the round morphology and appropriate cytoskeleton tension in accordance with physiological conditions, thus highlighting the need to establish such a platform to probe the interactions between chondrocytes and their niches. Taken together, this work not only shows the potential for the Mid-G/M ALG/COL hydrogel as a suitable matrix to promote cartilage formation, but it also implicates the biomechanical control of cell behavior in cell-based cartilage regeneration.

## Data Availability

The data that support the findings of this study are available upon reasonable request from the corresponding authors.
